# A case of radical surgery for primary lung cancer with idiopathic dilatation of the pulmonary artery: a case report

**DOI:** 10.1186/s44215-025-00193-7

**Published:** 2025-03-19

**Authors:** Wataru Shigeeda, Makoto Tomoyasu, Naoki Yanagawa, Hiroyuki Deguchi, Yuka Kaneko, Ryuuichi Yoshimura, Hironaga Kanno, Mayu Sugai, Shunsuke Shikanai, Hajime Saito

**Affiliations:** 1 Department of Thoracic Surgery, Iwate Medical University, 2-1-1, Idaidori, Yahaba, Shiwa, Iwate 028-3695 Japan; 2 Department of Molecular Diagnostic Pathology, Iwate Medical University, Iwate, Japan; 3 Department of Pulmonary Medicine, Hachinohe Red Cross Hospital, Aomori, Japan

**Keywords:** Lung cancer, Idiopathic dilatation of the pulmonary artery, Video-assisted thoracic surgery

## Abstract

**Background:**

The incidence rate of idiopathic dilatation of the pulmonary artery (IDPA) has been reported as 0.007%. We performed radical pulmonary resection under 3-port video-assisted thoracic surgery (VATS) in a patient with primary lung cancer and IDPA.

**Case presentation:**

A 61-year-old man presented with an abnormality identified on chest X-ray during a medical check. Computed tomography revealed a 56-mm pulmonary tumor (diagnosed as squamous cell carcinoma by bronchoscopy) located in S^6^ of the right lower lobe, with clinical middle and lower interlobular lymph node metastasis on the basis of lymphadenopathy. Moreover, the right and left main pulmonary arteries were dilated, but the common basal artery after branches A^4^, A^5^, and A^6^ was normal. The presence of pulmonary hypertension was ruled out by cardiac catheterization, which revealed a pulmonary artery pressure within the normal range (24/11 mmHg). No infectious disease was present, and the patient did not have any history of chronic inflammatory disease. Therefore, IDPA was diagnosed. Surgery was performed, and the intraoperative findings were consistent with those of preoperative CT. Although there were concerns that the pulmonary artery wall might have been weakened due to IDPA, all pulmonary arteries, including the dilated right intermediate trunk, were cut safely with a stapler in this case.

**Conclusion:**

In this case, the intraoperative and histopathological findings demonstrated no fragility of the pulmonary arteries, and the pulmonary artery was safely dissected using a stapler in 3-port VATS. However, radical surgery for lung cancer with IDPA is rare, and the safety needs to be verified by accumulating of further cases in the future.

## Background

Idiopathic dilatation of the pulmonary artery (IDPA) is very rare [1]. We performed radical pulmonary resection under 3-port video-assisted thoracic surgery (VATS) in a patient with primary lung cancer and IDPA. No previous reports have described surgery for primary lung cancer with IDPA, let alone 3-port VATS. We report a rare case of surgical resection for primary lung cancer with IDPA, including pathological findings.

## Case presentation

**Fig. 1:**
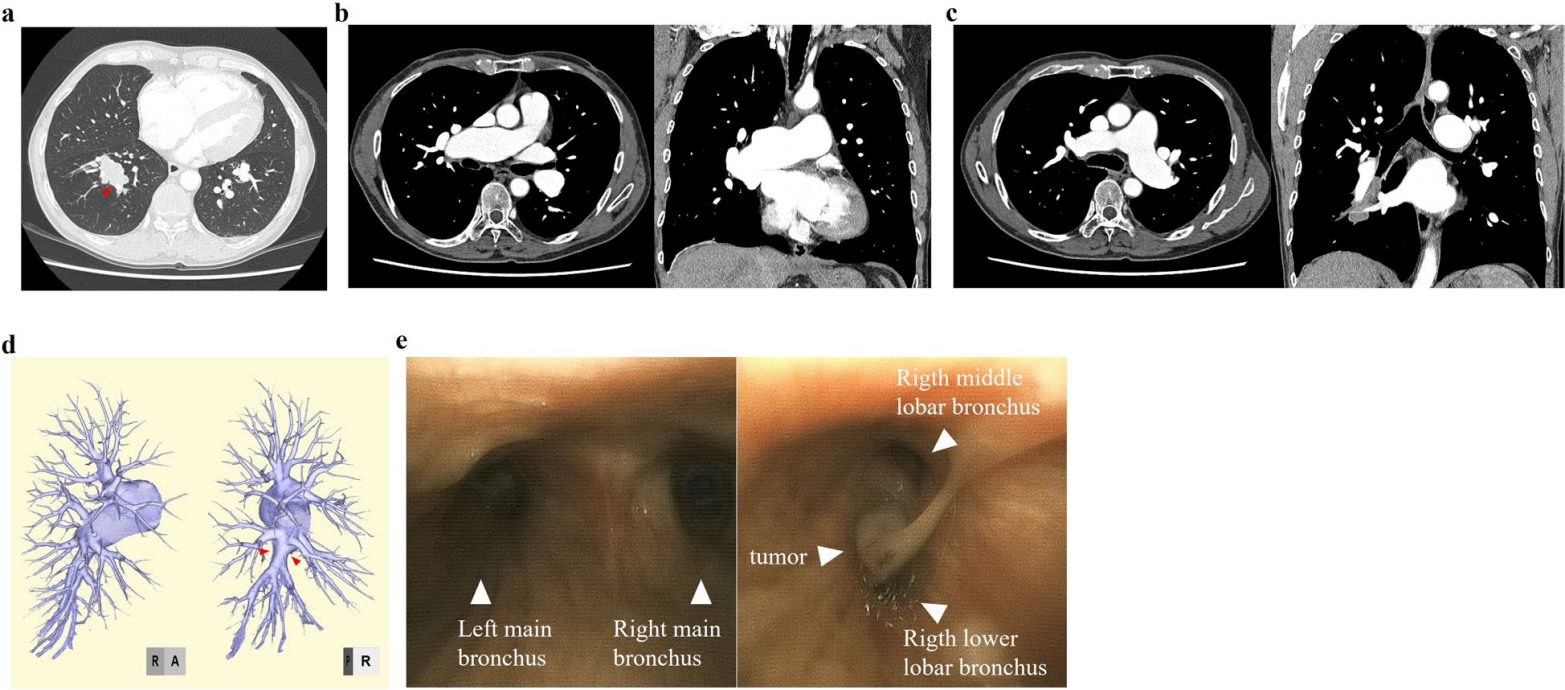
Preoperative examination images. **a** A mass in the right lower lobe on computed tomography (CT) (arrowhead). Dilation of the right main pulmonary artery (**b**) and left main pulmonary artery (**c**) on CT. **d** Pulmonary artery on 3DCT. The peripheral intermediate pulmonary artery is normal in size. **e** Bronchoscopy of the lower end of the trachea and intermediate bronchus

A 61-year-old man presented with an abnormality identified on chest X-ray during a medical check. He was asymptomatic, but had a medical history of hypertension, dyslipidemia, chronic obstructive pulmonary disease, and dilatation of the pulmonary artery. Computed tomography (CT) revealed a 56-mm pulmonary tumor located at S^6^ of the right lower lobe (Fig. 1a). Various preoperative was examined to facilitate surgery. The vital signs were normal except for blood pressure (141/77 mmHg). CT examination revealed dilated right (44 mm) and left (32 mm) main pulmonary arteries (Fig. 1b, c). In the right pulmonary artery, the maximum diameter of the intermediate pulmonary artery after it branches from the upper trunk artery was 31 mm, indicating considerable enlargement. On the other hand, the maximum diameter of the common basal artery and the A^4^, A^5^, and A^6^ branches was 10 mm, which was equivalent to the normal size (Fig. 1d).

Several were examined to determine the cause of pulmonary artery dilation. Transthoracic echocardiography showed an estimated right ventricular pressure of 34 mmHg, which was indicative of mild pulmonary hypertension, but no valvular disease of the heart. Cardiac catheterization revealed a pulmonary artery pressure of 24/11 mmHg. This value was within the normal range for pulmonary arterial pressure; the presence of pulmonary hypertension was ruled out. No infectious diseases such as tuberculosis, syphilis, or human immunodeficiency virus infection were identified in blood tests. In addition, the patient did not have any history of chronic inflammatory diseases such as rheumatoid arthritis, Behcet’s disease, or vasculitis. On the basis of these findings, we concluded that pulmonary artery dilation was not secondary to dilation caused by other diseases, but rather that IDPA developed in the absence of any clear triggers.

Bronchoscopy showed the tumor was seen to have spread beyond the orifice of the middle lobar bronchus, and diagnosed as squamous cell carcinoma on biopsy. There was no finding of narrowing of the trachea-bronchus due to external compression by the dilated vessels (Fig. 1e). CT showed a 56-mm mass expanding toward the central region of the middle lobar bronchus. In addition, clinically middle and lower interlobular lymph node metastasis was suspected based on the presence of lymphadenopathy. Therefore, we decided to perform bilobectomy (middle and lower).

The surgery started with 3-port VATS. The right pulmonary artery had significantly expanded proximal to the common basal artery, but the vessel distal to this artery showed no expansion, consistent with the preoperative CT findings. No thinning of the pulmonary artery was evident on gross inspection, so we continued radical surgery with 3-port VATS. First, A^6^, which showed a normal outer diameter, was cut off with a stapler (Endo GIA™ Tri-staple™ Gray cartridge 45 mm; Medtronic, Dublin, Ireland) including the purpose of confirming the structure of the pulmonary artery wall. No oozing from the cut edge was observed. There was almost no separation between the upper lobe and the middle lobe. Consequently, it was necessary to divide A^4^ and A^5^ individually before the transection of the intermediate pulmonary artery to ensure appropriate dissection margin. As the surgery was performed 3-port VATS, in order to divide the A^4^ at the back, it was necessary to divide the A^5^ first to secure the field of view and working space. As with A^6^, both A^4^ and A^5^ were cut off using the same stapler (Endo GIA™ Tri-staple™ Gray cartridge 45 mm), and no problems with either cutting edge were observed. At this point, we concluded that there were no major problems with the structure of the pulmonary artery wall, and cutting with a stapler was deemed safe.

**Fig. 2:**
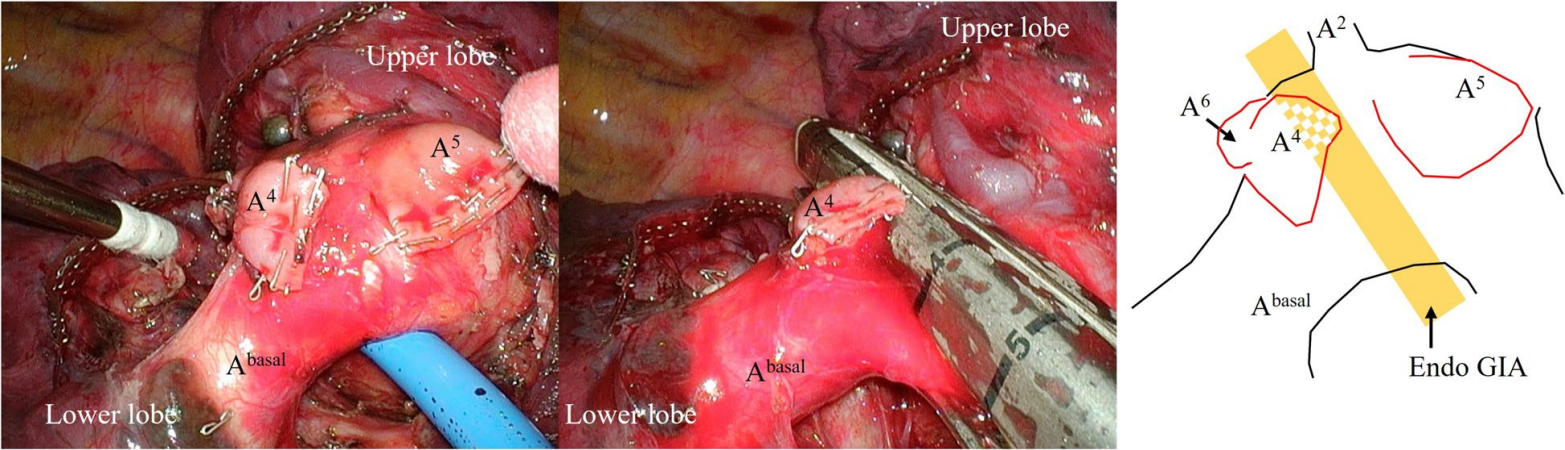
Intraoperative images. The dilated intermediate pulmonary artery is cut off with an Endo GIA™ Tri-staple™ Camel cartridge 60 mm after first cutting off A^4^, A^5^, and A^6^ separately. The schematic illustration shows the relative positions of the pulmonary artery, and the position of the line that was the intermediate pulmonary artery was cut off

In preparation for any eventuality, the main right pulmonary artery was dissected to allow immediate clamping and maintenance in a free state. The dilated intermediate pulmonary artery was subsequently cut off with a larger stapler (Endo GIA™ Tri-staple™ Camel cartridge 60 mm; Medtronic) (Fig. 2). The ideal approach would have been to divide the intermediate pulmonary artery proximal to the dividing A^5^, but because the A^2^ branched from the pulmonary artery on the opposite side of the same level as A^5^, it was not possible to divide the middle pulmonary artery proximal to the A^5^. Therefore, we chose to divide the intermediate pulmonary artery between the A^4^ and A^5^. All cutting with the staplers was safely performed without oozing from the cut edge or vessel rupture. Bilobectomy and lymph node dissection were completed under 3-port VATS. No complications occurred during the perioperative period, and the patient was discharged on postoperative day 7.

In cases of secondary dilatation of the pulmonary artery, the pulmonary artery is known to become vulnerable by the thinning of the vessel walls or the snapping apart of elastic fibers [2]. Therefore, our primary concern was whether the pulmonary artery burst after being cut off due to a weakened vessel wall. In this case, the three pulmonary arteries were cut off before the intermediate pulmonary artery was cut off, and the cross section of the arteries did not appear markedly different from those in patients without IDPA. Therefore, we chose to use a stapler to cut off the intermediate pulmonary artery and were able to safely complete the surgery.

**Fig. 3:**
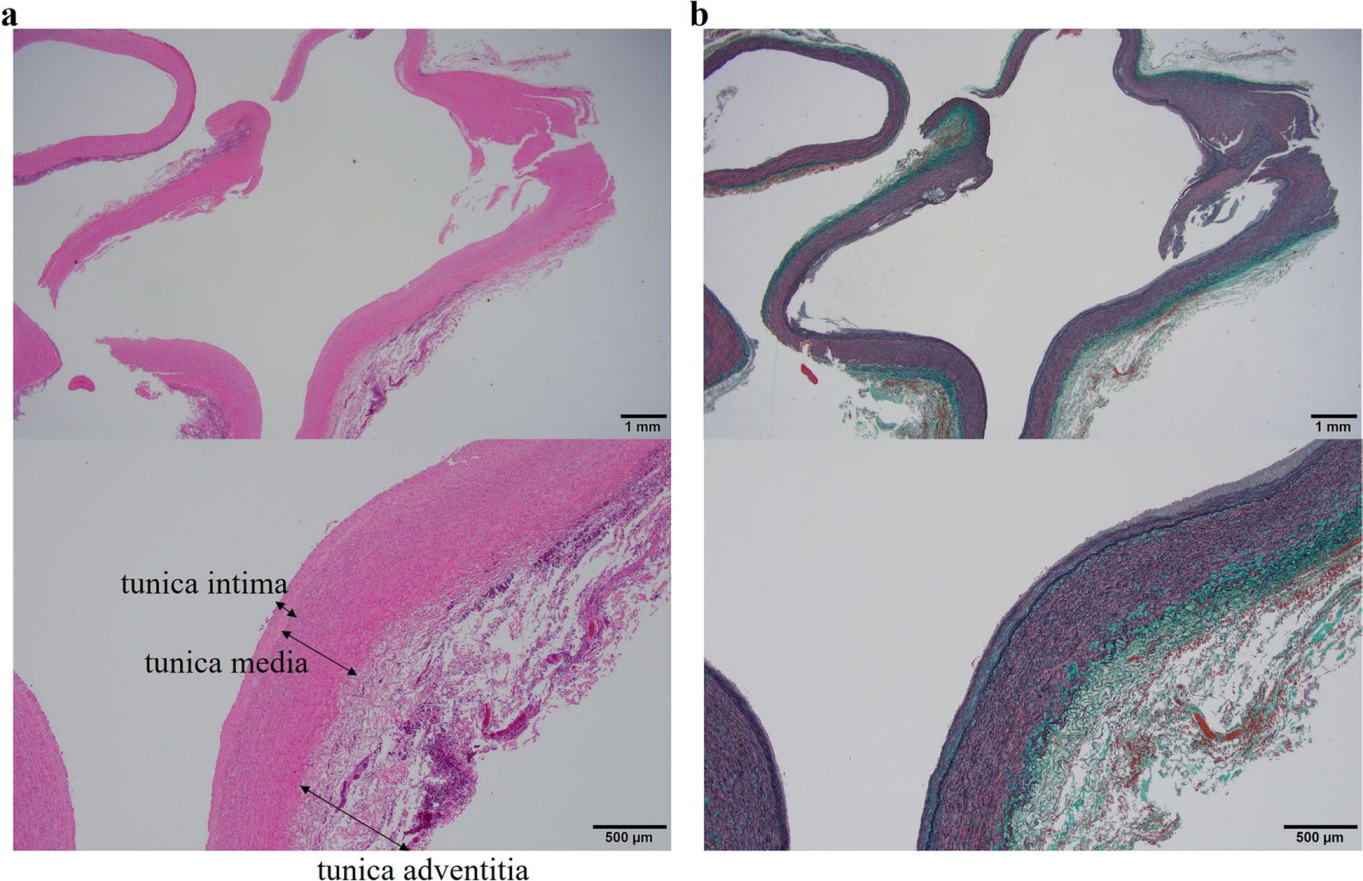
Histopathological images. Histopathological findings of dilated intermediate pulmonary artery walls with hematoxylin and eosin staining (**a**) and elastic Masson staining (**b**) at magnifications of 12.5 × and 100 × , respectively

**Fig. 4:**
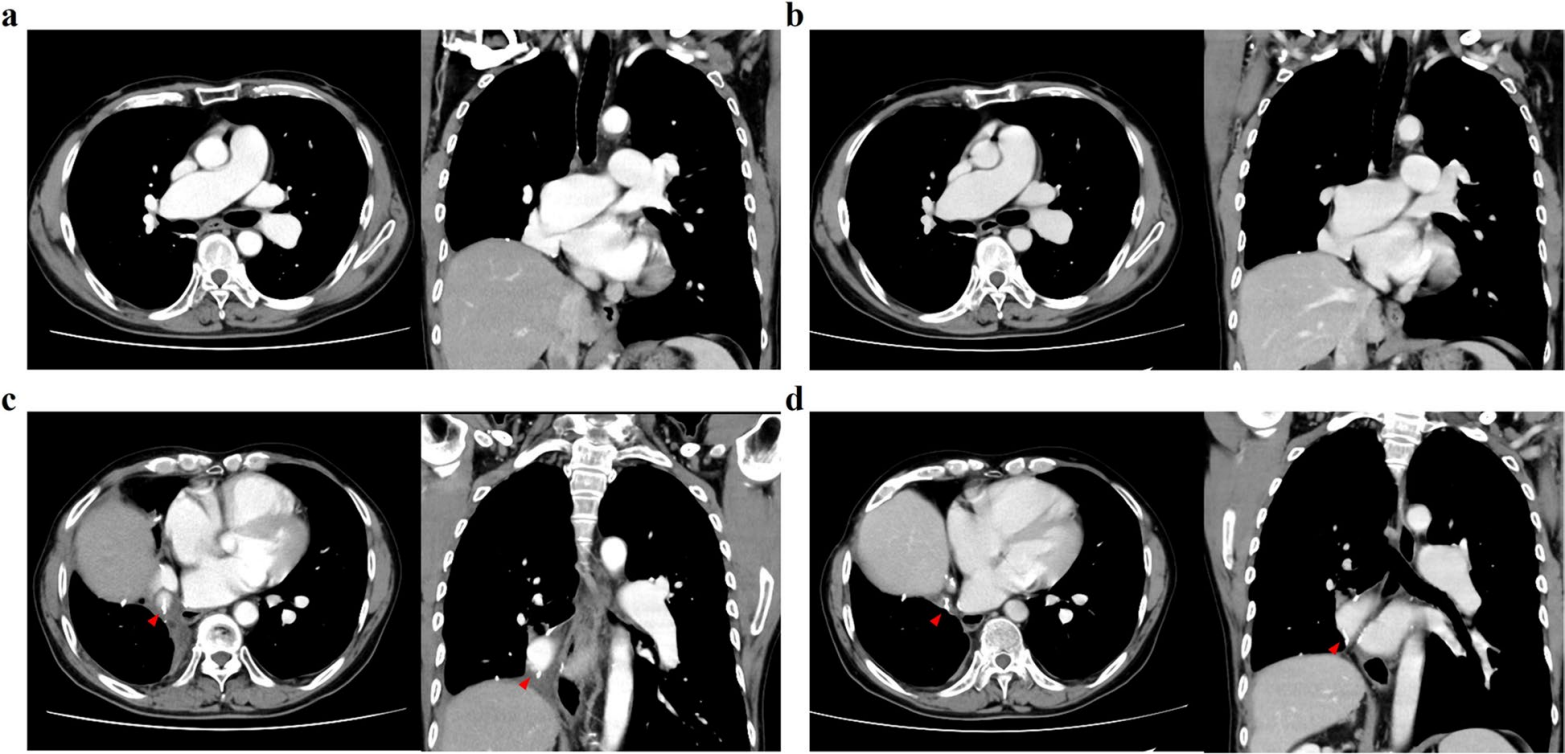
Preoperative CT images. The dilated right main pulmonary artery at 6 months (**a**) and 2 years (**b**) after surgery. The position of cut off the dilated intermediate pulmonary artery 6 months (**c**) and 2 years (**d**) after surgery

The pathological result was keratinizing squamous cell carcinoma. The tumor size was 35 × 30 × 25 mm, and one of the intrapulmonary metastases was shown in right lower lobe. The evaluation of the lymph nodes showed that the middle and lower interlobular lymph nodes that appeared swollen on CT was metastasis. No metastases were found in the other lymph nodes. Histological examination of the edge of the right intermediate pulmonary artery was also performed (Fig. 3). At first glance, the blood vessel walls appeared thicker. However, the ratio of vessel wall thickness to vessel inner diameter was almost the same as what would be expected in a patient without IDPA. In addition, the vessel wall did not show any abnormal findings such as thickening of the tunica media or snapping apart of elastic fibers under hematoxylin and eosin or elastic Masson staining. CT scans performed 6 months and 2 years after the operation (Fig. 4a, b) show that dilation of the right main pulmonary artery did not change. The dilated intermediate pulmonary artery was cut off at the position of the staple lines (arrowheads) (Fig. 4c, d). There were no significant changes in the maximum diameter 6 months and 2 years after the operation.

## Discussion and conclusions

IDPA was first reported in 1923 by Wessler and Jaches [2]. The incidence of IDPA has been reported to be 0.007% [1]. Sharma et al. [3] stated that all of the following five items are necessary as diagnostic criteria for IDPA. (1) Simple dilatation of the pulmonary trunk with or without involvement of the rest of arterial tree. (2) Absence of intra-cardiac or extracardiac shunts. (3) Absence of chronic cardiac or pulmonary disease. (4) Absence of arterial disease, such as syphilis, or more than minimal atheromatosis or arteriosclerosis of the pulmonary vascular tree. (5) Normal pressure in the right ventricle and right ventricle. IDPA is often treated as a cardiovascular disease, and almost no pathological knowledge of this disease has been acquired [4]. Among the various autopsy reports, cases in which a dilated pulmonary artery was incidentally recognized have been described [1]. No previous reports have described surgery for primary lung cancer with IDPA.

In this surgical case, our primary concern was whether the pulmonary artery burst after being cut off because of a weakened vessel wall. In cases of secondary dilatation of the pulmonary artery in patients with Marfan syndrome [5] or pulmonary hypertension, the pulmonary artery becomes vulnerable. The reason was reported to involve thinning of the vessel walls or the snapping a part of elastic fibers [6]. This is the same reason why vascular replacement is necessary in patients who expand. No clear reason for dilatation of the pulmonary artery was identified in this patient because no underlying diseases, such as Marfan syndrome or pulmonary hypertension, were present. IDPA was therefore diagnosed. However, whether the pulmonary artery vessels were thinning or fragile was not known in advance because few reports have provided pathological findings from IDPA [7].

We routinely use the Endo GIA™ Tri-staple™ Gray cartridge, which corresponds to the thinnest tissue, to cut off the pulmonary artery. In this case, we wanted to select the Endo GIA™ Tri-staple™ Gray cartridge corresponding to the thinnest tissue, particularly to cut off the dilated intermediate pulmonary artery, as the pulmonary artery wall could have been thinning. However, the Endo GIA™ Tri-staple™ Gray cartridge was only 45 mm long, which was insufficient to allow the dilated intermediate pulmonary artery to be cut over its entire length. We therefore selected the Endo GIA™ Tri-staple™ Camel cartridge 60 mm, corresponding to a thicker organization. For safety reasons, the dilated intermediate pulmonary artery was cut off after dissecting the main right pulmonary artery so that it could be clamped immediately when a misfire occurred. Fortunately, the structures of the dilated intermediate pulmonary artery vessels in this patient did not significantly differ from those in patients without IDPA. On the other hand, although these are old reports, some have stated that the elastic fibers of pulmonary artery vessels are torn in patients with IDPA [8–10]. Moreover, it is impossible to know whether these cases actually represented secondary dilatation of the pulmonary artery or true IDPA caused by unrecognized mechanisms. In this case, the dilated intermediate pulmonary artery was cut off without securing the right main pulmonary artery with vascular tape, and it was possible to cut off it without any problems. However, in order to be even safer considering the possibility of rupture of the elastic fibers in the blood vessel wall, it might be necessary to not only dissect the right main pulmonary artery but also to secure it with vascular tape.

Andrews et al. reported a case of a 31-year-old man with IDPA who died suddenly [11]. The cause of death was pulmonary artery deviation and associated cardiac tamponade. In contrast, Sueta et al. reported a case of an 86-year-old woman with IDPA [7]. She eventually died of heart failure but experienced no cardiovascular symptoms before her death. Pathological examination showed a decrease in elastic fibers and an increase in collagen fibers. If these cases do indeed represent IDPA, then survival to a certain age without cardiovascular events may indicate negation of the vulnerability of the pulmonary arteries. Whether that age is 50 years, 60 years, or more cannot yet be discussed properly because the number of cases is small. However, the common age at onset of primary lung cancer is 65 years or older in Japan [12], and patients who have not experienced cardiovascular events until later ages may have no problems with stapler dissection of the pulmonary artery, as in normal patients.

In this case, the pulmonary artery was safely dissected using a stapler in 3-port VATS. No fragility of the pulmonary arteries was observed on intraoperative and histopathological findings. However, there are also reports that IDPA is associated with ruptures in the elastic fibers of the main pulmonary artery [4]. In the case of cut off the more central artery, for example, the upper lobectomy, there is no guarantee that the pulmonary artery will not be found to be fragile. Therefore, radical surgery for lung cancer patients with IDPA is rare, and the safety needs to be verified through the accumulation of further cases in the future.

## Data Availability

The data supporting the conclusions of this article are included within the article.

## Abbreviations

CT: Computed tomography
IDPA: Idiopathic dilatation of the pulmonary artery
VATS: Video-assisted thoracic surgery
